# [Retracted] Metformin upregulates E-cadherin and inhibits B16F10 cell motility, invasion and migration

**DOI:** 10.3892/ol.2026.15936

**Published:** 2026-11-03

**Authors:** Guanzhao Liang, Minglei Ding, Haitao Lu, Na Cao, Yandong Niu, Yang Gao, Jie Lu

Oncol Lett 10: 1527-1532, 2015; DOI: 10.3892/ol.2015.3475

Following the publication of the above paper, it has been drawn to the Editor's attention by a concerned reader that the data panels showing the results of invasion assay experiments in Fig. 3A on p. 1530 contained a number of overlapping sections of data, such that these panels, which were intended to show the results of four different experimental conditions, had all apparently been derived from the same original source. In addition, regarding the western blots shown in Fig. 4B, the protein bands shown for the E-cadherin experiments in the pair of lanes on the left in this figure part looked remarkably similar, suggesting that this protein band may have been duplicated within the gel.

Given that these issues have come to light, the Editor of *Oncology Letters* has decided that this paper should be retracted from the Journal on account of a lack of confidence in the presented data. After contacting the authors, they accepted the decision to retract the paper. The Editor apologizes to the readership for any inconvenience caused.

